# COVID-19 and arrhythmia: The factors associated and the role of myocardial electrical impulse propagation. An observational study based on cardiac telemetric monitoring

**DOI:** 10.3389/fcvm.2022.912474

**Published:** 2022-09-07

**Authors:** Domenico Cozzolino, Ciro Romano, Riccardo Nevola, Aldo Marrone, Giuseppina R. Umano, Giovanna Cuomo, Luca Rinaldi, Luigi E. Adinolfi, Vanvitelli COVID Collaborators, Abitabile Marianna

**Affiliations:** Aprea Concetta, Cirigliano Giovanna, and Ruocco Rachele; ^1^Department of Precision Medicine, University of Campania Luigi Vanvitelli, Naples, Italy; ^2^Department of Advanced Medical and Surgical Sciences, University of Campania Luigi Vanvitelli, Naples, Italy; ^3^Department of the Woman, the Child, of General and Specialized Surgery, University of Campania Luigi Vanvitelli, Naples, Italy

**Keywords:** COVID-19, arrhythmia, QT interval, cardiac telemetry, QT dispersion

## Abstract

**Background:**

The heart is commonly involved in COVID-19, and rhythm disorders have been largely reported.

**Objective:**

To evaluate the association of some non-cardiac and cardiac comorbidities and QT dispersion with arrhythmias and their impact on outcomes in hospitalized patients with COVID-19.

**Methods:**

Each patient underwent cardiac telemetry monitoring through the entire hospitalization period, laboratory analyses, 12-lead ECG, and lung imaging examination. Patients with arrhythmia were divided into three groups (bradyarrhythmias, tachyarrhythmias, and tachy- and bradyarrhythmias).

**Results:**

Two-hundred patients completed the study (males, 123; mean age, 70.1 years); of these, 80 patients (40%) exhibited rhythm disorders on telemetry. Patients with arrhythmia were older (*p* < 0.0001), had a greater number of comorbidities (*p* < 0.0001), higher values of creatinine (*p* = 0.007), B-type natriuretic peptide (*p* < 0.0001), troponin (*p* < 0.0001), C-reactive protein (*p* = 0.01), ferritin (*p* = 0.001), D-dimer (*p* < 0.0001), procalcitonin (*p* = 0.0008), QT interval (*p* = 0.002), QTc interval (*p* = 0.04), and QTc dispersion (*p* = 0.01), and lower values of sodium (*p* = 0.03), magnesium (*p* = 0.04), glomerular filtration rate (*p* < 0.0001), and hemoglobin (*p* = 0.008) as compared to patients without arrhythmia. By comparing the three subgroups of patients, no significant differences were found. At multivariate analysis, age [odds ratio (OR) = 1.14 (95% CI: 1.07–1.22); *p* = 0.0004], coronary artery disease [OR = 12.7 (95% CI: 2.38–68.01); *p* = 0.005], and circulating troponin [OR = 1.05 (95% CI: 1.003–1.10); *p* = 0.04] represented risk factors independently associated with arrhythmia. All-cause in-hospital mortality was ∼40-fold higher among patients with arrhythmia [OR = 39.66 (95% CI: 5.20–302.51); *p* = 0.0004].

**Conclusion:**

Arrhythmias are associated with aging, coronary artery disease, subtle myocardial injury, hyperinflammatory status, coagulative unbalance, and prolonged QTc dispersion in patients with COVID-19, and confer a worse in-hospital prognosis. Given its usefulness, routinary use of cardiac telemetry should be encouraged in COVID wards.

## Introduction

Severe acute respiratory syndrome coronavirus 2 (SARS-CoV-2) infection, responsible for coronavirus disease 2019 (COVID-19), was first reported in Hubei Province, China at the end of 2019. Apart from its serious social and economic consequences, this worldwide disease is characterized by high mortality; to date, a total of about 5.5 million deaths secondary to acute respiratory distress syndrome and/or damage to other organs/systems have been documented. In particular, cardiovascular manifestations have been recognized as one of the most common complications among patients hospitalized with the disease. Previous studies conducted in hospitalized patients with COVID-19 from China showed a significant elevation of high-sensitive troponin I in about 25% of patients and a higher mortality rate (about 10-fold) as compared to those with no heart injury (1, 2). Moreover, a cardiac MR-based study revealed a subtle heart involvement in up to almost 80% of patients with COVID-19, independently of preexisting cardiac conditions and irrespectively of course and gravity of viral disease (3).

As expected, a higher risk for fatal and/or non-fatal arrhythmias has been reported among patients with COVID-19 with or without manifest cardiac involvement (4, 5). Conceptually, several are the mechanisms potentially leading to arrhythmias in SARS-CoV-2 infection, including electrical imbalance secondary to direct myocyte injury, hypoxia due to lung disease and/or pulmonary embolism, drugs, and, finally, intravascular volume and electrolyte disequilibrium secondary to direct gastrointestinal and kidney virus-related derangement. As already reported, previous and/or concomitant arrhythmic episodes have been found to negatively influence in-hospital prognosis in patients with COVID-19 (5).

In addition, secondary to SARS-CoV-2-related myocyte injury could play a potential proarrhythmic role in patients with COVID-19. Prolongation of QT interval, as a consequence of both a direct or indirect action of some drugs on myocardial cells (for example, chloroquine and hydroxychloroquine often administered in such patients) and electrolyte imbalance due to the therapeutic use of corticosteroid and diuretic drugs, could represent a potential for arrhythmia in patients affected by COVID-19. Indeed, a prolonged QT duration has been recognized to be associated with severe rhythm disorders leading in some cases to death, such as torsade de pointes (6).

Interlead differences in QT interval duration on standard 12-lead ECG are known as QT dispersion. A condition of damage to myocardial tissue secondary, for example, to inflammation and/or hypoxia may be responsible for abnormal propagation of electrical impulse, which, in turn, accounts for regional heterogeneity of QT duration. Increased values of QT dispersion have been reported to be associated with a higher risk of fatal and non-fatal arrhythmias in humans (7–9). To date, few are available data on the eventual association between duration and dispersion of absolute and heart rate-corrected QT (QTc) interval and the risk of rhythm disturbances in patients affected by COVID-19.

The present study was conducted in a cohort of patients with COVID-19 consecutively admitted and closely monitored through the entire hospitalization period by means of cardiac telemetry. The present study aimed to evaluate prospectively the main non-cardiac comorbidities and some cardiac conditions, including QTc dispersion, likely associated with rhythm disorders among patients with COVID-19. Moreover, the present study aimed to provide a further contribution to exploring a potential predictive role of arrhythmia on in-hospital outcomes in this setting of patients.

## Materials and methods

We conducted a prospective study including all the consecutive patients with COVID-19 referring to the COVID Center at the University of Campania Luigi Vanvitelli, Naples, Italy, between 15 December 2020 and 15 May 2021. All the patients exhibited positive PCR testing for SARS-CoV-2, and all showed pulmonary involvement as confirmed by imaging tests, such as lung X-ray, and/or tomography scans, and/or ultrasonography. All the patients were hospitalized during the acute phase of illness and, therefore, all of them were closely monitored through the entire hospitalization period. Patient’s history, physical examination, and laboratory and instrumental investigations were collected on in-hospital admission day. The severity of illness was judged on the basis of pulmonary involvement degree, which was established by using two conventional scores: lung CT score (LCTS) (10), and lung ultrasound score (LUS) (11). Briefly, the LCTS is a semiquantitative method used to describe the involvement degree of each of the five lung lobes, so that a total score ranged from 0 (no involvement) to 25 (maximum involvement). The LUS was acquired on the standard sequence of 14 peculiar anatomic landmarks with a score ranging between 0 and 3 and is based on impairment of ultrasound picture; the total score was calculated by their sum and ranged from 0 (no involvement) to 42 (maximum involvement).

### Electrocardiography

Each patient received a 12-lead ECG both on admission day and at discharge. ECG was recorded at a standard speed of 25 mm/s. The QT interval was measured from the beginning of the QRS complex to the end of the T wave. The point at which the T wave returns to baseline is defined as its end; the U wave, if present, was excluded from the QT calculation. In patients with bundle branch block, the QT interval was measured according to a formula described by Bogossian; practically, the modified QT (QTm) in patients with bundle branch block or with a pacemaker was estimated using the following formula: QTm = QT − 48.5% QRS (12). The data were excluded from the study if the T wave was not reliably measurable. QT dispersion was defined as the difference between the maximum and the minimum QT intervals for any of the 12 leads on ECG trace in each patient. The Fridericia formula was utilized to calculate QTc: QTc = QT/cube root of the RR interval (13). Echocardiographic scans were performed where necessary.

### Telemetry monitoring

Electrocardiogram trace was continuously monitored during the entire period of hospitalization in all the patients by 7-lead ECG telemetry (Dräger Vista 120 S, Drägerwerk AG, Lübeck, Germany). On the basis of ECG trace analysis, it was possible to identify patients with and without arrhythmia. Patients with arrhythmia were divided into the three groups: (1) with bradyarrhythmias (B subgroup), i.e., evidence of sinus bradycardia (<60 beats/min), and/or conduction disturbances [including sinoatrial and atrioventricular block (second degree or higher)]; (2) with tachyarrhythmias (T subgroup), i.e., evidence of regular supraventricular tachycardia, and/or atrial fibrillation [according to the European Society of Cardiology (ESC) guidelines (14), defined as minimum duration of more than 30 s], and/or atrial flutter, and/or frequent premature ventricular contractions when significant (defined as > 10 per min or > 30 per h) (15), and/or ventricular tachycardia (non-sustained and sustained, defined as three or more consecutive premature ventricular complexes at a rate of >100 beats/min and lasting less or more than 30 s, respectively), and/or polymorphic ventricular tachycardia (torsade de pointes), and/or ventricular fibrillation; and; (3) with both brady- and tachyarrhythmias (BT subgroup). ECG traces on cardiac telemetry were interpreted by two experienced cardiologists blinded to patients’ data.

### Laboratory assays

Laboratory measurements were performed inside our institution by available commercial kits.

### Statistics

Continuous variables were expressed as median [interquartile range (IQR)] according to their distribution, and categorical variables were expressed as frequencies. Continuous variables were checked for normality with the Kolmogorov–Smirnov test. Differences for continuous variables were investigated with the Student’s *t*-test for independent samples and the Mann–Whitney *U* test as appropriate. The chi-squared tests were performed for differences in categorical variables. *Post-hoc* analysis with Bonferroni correction for multiple comparisons has been performed when appropriate. Variables, which demonstrated association (*p* < 0.05) with arrhythmia after comparison of characteristics of the patients with and without arrhythmia, were included in the univariate analyses. The univariate logistic regression analyses were performed to identify candidate variables for multivariate analysis to estimate the risk of presenting arrhythmia. Statistically significant variables in the univariate analysis have been included in the multivariate analysis. All the analyses have been performed using SAS^®^ University Edition (SAS Institute Incorporation, Cary, NC, United States). *P*-values < 0.05 were considered statistically significant.

### Study approval

The study was conducted in accordance with the Declaration of Helsinki and approved by the Ethics Committee of the University of Campania Luigi Vanvitelli.

## Results

Our cohort of patients with COVID-19 consisted of 208 individuals; males were 128 (61.5%) and the mean age was 70.1 years (range: 26–94 years). Based on patients’ personal history and physical examination, it resulted that 49 patients (23.6%) were affected by obesity, 50 patients (24.0%) were affected by diabetes, 55 patients (26.4%) were affected by cardiovascular diseases, and 114 patients (54.8%) were affected by systemic arterial hypertension. The average number of comorbid conditions was 2.8. In-hospital mortality rate was 10.1% (*n* = 21; M/F, 15/6). The mean age of the deceased patients was 77.3 years (range: 61–93 years), and their mean number of comorbidities was 3.2.

Electrocardiogram trace on telemetry was available and exhaustively analyzed in 200 of 208 individuals; 8 patients were excluded from analysis because of one of the following: intolerance to ECG monitoring instrumentation (*n* = 3), important skin reaction to ECG gel/electrodes (*n* = 2), and poor interpretability of ECG traces (*n* = 3). None among these eight excluded patients deceased.

### Patients without arrhythmia

These individuals (*n* = 120; 60%) did not show significant rhythm disorders during the entire period of hospitalization. Among this patients group, one patient (0.8%) died. The cardiovascular profile of these patients is given in Table 1. Among these subjects, 12 patients (10%) complained of prior arrhythmias and were on antiarrhythmic treatment as priorly scheduled. The mean number of comorbid conditions was 1.8. LCTS and LUS were 8.9 + 3.8 (mean + SD) and 14.7 + 7.6, respectively. By analyzing the ECG trace, values of the main parameters (i.e., QT, QTc, and QTc dispersion) on admission day were not significantly different when compared to those recorded at discharge (Table 2).

**TABLE 1:** Characteristics of patients monitored on cardiac telemetry.

	Without arrhythmia (*n* = 120)	With arrhythmia (*n* = 80)	*p-*value
Age (years)	65 (57.5–72.5)	74.5 (68–80.5)	<0.0001
Sex (M/F)	72/48	51/29	0.29
Obesity n (%)	30 (25)	18 (22.5)	0.69
Diabetes mellitus n (%)	26 (21.7)	24 (30)	0.18
Chronic liver disease n (%)	14 (11.7)	9 (11.3)	0.93
Chronic kidney disease n (%)	8 (6.7)	14 (17.5)	0.02
Chronic obstructive pulmonary disease n (%)	12 (10.0)	19 (23.7)	0.008
Current tobacco n (%)	52 (43.3)	38 (47.5)	0.56
Hyperlipidemia n (%)	17 (14.2)	8 (10)	0.38
Autoimmune disease n (%)	10 (8.3)	6 (7.5)	0.83
Active malignancy n (%)	5 (4.2)	3 (3.7)	0.88
Prior organ transplantation n (%)	0 (0)	4 (5)	–
Rare disease n (%)	3 (2.5)	7 (8.8)	0.047
Arterial hypertension n (%)	59 (49.2)	55 (68.7)	0.006
Coronary artery disease n (%)	7 (5.8)	22 (27.5)	<0.0001
Heart failure n (%)	1 (0.8)	12 (15)	<0.0001
Any previous arrhythmia n (%)	12 (10)	34 (42.5)	<0.0001
ICD/PPM n (%)	0 (0)	10 (12.5)	–
Comorbidities	3 (1–4)	3 (2–4)	<0.0001
**Instrumental/Laboratory findings**			
Left ventricle ejection fraction (%)	57 (54–60)	53 (50–57)	0.53
Sodium (mmol/l)	138 (136–140)	137 (135–140)	0.03
Potassium (mmol/l)	4.2 (3.9–4.5)	4.2 (3.8–4.6)	0.99
Magnesium (mmol/l)	2.1 (1.9–2.2)	1.9 (1.8–2.1)	0.04
Calcium (mg/dl)	8.7 (8.4–9.0)	8.7 (8.4–9.0)	0.81
Creatinine (mg/dl)	0.80 (0.72–0.87)	0.93 (0.70–1.17)	0.007
Glomerular filtration rate (ml/min)	94.5 (79.3–103.0)	72.1 (49.0–89.1)	<0.0001
B-type natriuretic peptide (pg/ml)	11.0 (8.0–25.0)	84.5 (24.0–221.0)	<0.0001
Troponin (ng/ml)	2.0 (2.0–5.0)	10.0 (4.0–27.8)	<0.0001
C-reactive protein (mg/dl)	3.93 (1.44–8.69)	6.24 (2.57–12.50)	0.01
Ferritin (ng/ml)	398.0 (208.5–911.5)	844.5 (479.0–1175.0)	0.001
Interleukin-6 (pg/ml)	12.6 (8.2–30.0)	30.0 (22.7–41.5)	0.06
D-dimer (ng/ml)	400 (200–760)	830 (350–1440)	<0.0001
Procalcitonin (ng/ml)	0.05 (0.02–0.10)	0.11 (0.03–0.39)	0.0008
White blood cells (cells/μl)	7,340 (5,600–9,655)	8,040 (4,850–11,570)	0.40
Hemoglobin (g/dl)	13.8 (12.8–14.8)	13.1 (9.7–14.2)	0.008
**In-hospital medication**			
Azithromycin n (%)	17 (14.2)	14 (17.5)	0.52
Quinolones n (%)	10 (8.3)	7 (8.8)	0.91
Haloperidol n (%)	3 (2.5)	3 (3.7)	0.61
Remdesivir n (%)	50 (41.7)	26 (32.5)	0.19
Tocilizumab n (%)	8 (6.7)	10 (12.5)	0.16
Hydroxychloroquine n (%)	3 (2.5)	3 (3.7)	0.61
Dexamethasone n (%)	84 (70)	61 (76.3)	0.33
Low-molecular-weight heparin n (%)	114 (95)	75 (93.7)	0.77

Values are presented as median (interquartile range) or as n (%). ICD/PPM, implantable cardioverter-defibrillator/permanent pacemaker.

**TABLE 2:** Type of arrhythmia recorded on cardiac telemetry during in-hospital period.

Tachyarrhyhtmias	(n)
**Supraventricular**	
Non-sustained regular atrial tachycardia	9
New onset non-sustained regular atrial tachycardia	9
Sustained regular atrial tachycardia	0
New onset sustained regular atrial tachycardia	5
Atrial fibrillation	11
New onset atrial fibrillation	7
Atrial flutter	0
New onset atrial flutter	2
**Ventricular**	
Premature ventricular complexes	14
New onset ventricular complexes	19
Non-sustained ventricular tachycardia	2
New onset non-sustained ventricular tachycardia	7
Sustained ventricular tachycardia	0
New onset sustained ventricular tachycardia	1
Polymorphic ventricular tachycardia	0
Ventricular fibrillation	1

**Bradyarrhythmias**	
Sinus bradycardia	7
New onset sinus bradycardia	16
Sinoatrial block (II degree or higher)	0
New onset sinoatrial block (II degree or higher)	2
Conduction disturbances (II degree or higher AVB, and/or BBB)	17
New onset conduction disturbances (II degree or higher AVB, and/or BBB)	3

AV, atrioventricular block; BBB, bundle branch block.

### Patients with arrhythmia

Altogether, 80 patients (40%) showed the ECG trace suggesting rhythm abnormalities on cardiac telemetry. Of these, 46 patients (57.5%) developed new-onset arrhythmias during the in-hospital period, whereas the remaining 34 patients were already and chronically suffering from arrhythmic disturbances, and all of them continued to be treated with their antiarrhythmic drugs as priorly scheduled. Among patients with arrhythmia, 20 of these subjects had died; overall in-hospital mortality among patients with arrhythmia was significantly (25 vs. 0.8%; *p* < 0.0001) higher than that among patients without arrhythmia. Furthermore, arrhythmia resulted associated with a significant increase in in-hospital all-cause mortality [odds ratio = 39.66 (95% CI: 5.20–302.51); *p* = 0.0004]. As given in Table 1, patients with arrhythmia were significantly (*p* < 0.0001) older, and showed a significantly (*p* < 0.0001) higher number of comorbid conditions as compared to patients without arrhythmia (Table 1). Serum sodium and magnesium levels, glomerular filtration rate, and hemoglobin values in patients with arrhythmia were significantly (*p* < 0.04, at least) lower than those in patients without arrhythmia, whereas serum creatinine, B-type natriuretic peptide (BNP) and troponin, and plasma levels of C-reactive protein, procalcitonin, and D-dimer were significantly (*p* < 0.01, at least) higher (Table 1). Serum levels of interleukin-6 in patients with arrhythmia were slightly but not significantly higher as compared to those without arrhythmia (Table 1). LCTS and LUS in patients with arrhythmia were 8.4 + 3.7 (mean + SD) and 15.4 + 7.5, respectively, and not significantly different from those in patients without arrhythmia. Use (namely, type and posology) of in-hospital medications was non-significantly different by comparing the two groups (Table 1).

**FIGURE 1:**
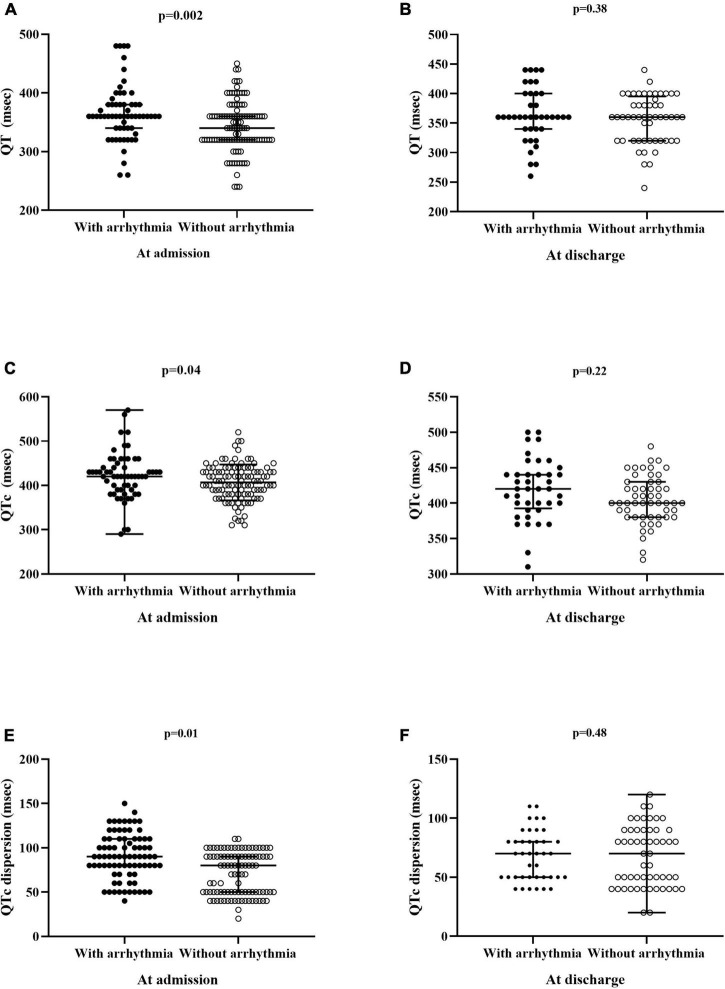
ECG parameters in patients with and without arrhythmia monitored on cardiac telemetry. **(A,C,E)** Data of patients (*n* = 200) at admission. **(B,D,F)** Data of patients (*n* = 179) at discharge. QTc, heart rate-corrected QT interval.

By analysis of ECG trace on admission day, it resulted that QT, QTc, and QTc dispersion values in patients with arrhythmia were significantly (*p* < 0.04, at least) higher when compared to those in patients without arrhythmia (Figure 1). At discharge, ECG parameters in patients with arrhythmia were not significantly different as compared to those measured in patients without arrhythmia (Figure 1). Paired intragroup comparisons of ECG parameters between those on admission day and those measured at discharge revealed that QTc dispersion values at discharge were significantly (*p* < 0.007) lower than those recorded on admission solely in the group of patients with arrhythmia (Figure 1). The type of arrhythmia recorded on telemetry during the entire hospitalization period is given in Table 2.

### Subgroups of patients with arrhythmia

The main general findings of patients and their main ECG parameters (recorded both on admission and at discharge) allocated in the subgroups B (12 patients), T (57 patients), and BT (11 patients) are shown in Table 3. *Post-hoc* analysis showed that QT values on admission in the BT subgroup were significantly (*p* = 0.03) higher as compared to those in the T subgroup. Paired comparison between ECG parameters calculated on admission and those at discharge showed no differences for QT and QTC in all the subgroups. Conversely, QTc dispersion values at discharge tended to slightly (*p* = 0.06) decrease in the B subgroup and to significantly (*p* = 0.047) decrease in the BT subgroup, as compared to admission day. Moreover, by comparing patients (*n* = 34) with previously diagnosed and current arrhythmias with those (*n* = 46) with new-onset arrhythmias, no statistically significant differences in the prevalence of the main comorbid cardiac diseases, degree of lung involvement, use of antiarrhythmic medication, ECG parameters, and in-hospital mortality rate were found (Table 4).

**TABLE 3:** General features, cardiovascular profile, and ECG measurements in the subgroups of patients with arrhythmia.

	B (*n* = 12)	T (*n* = 57)	BT (*n* = 11)	*p-*value
**General findings**				
Age (years)	64.5 (67–80.5)	79 (74–79)	79 (66–87)	0.89
Death n (%)	2 (16.7)	16 (28.1)	2 (18.2)	0.61
Comorbidities	3.5 (2–5)	3 (2–4)	3 (2–4)	0.44
Lung computed tomography score	8 (5–15)	8 (5–12)	8 (7–9)	0.96
Lung ultrasound score	8 (5–15)	13.5 (7–20)	11 (4–14)	0.48
**Main cardiovascular morbidities**				
Arterial hypertension n (%)	8 (66.6)	39 (68.4)	8 (72.7)	0.78
Coronary artery disease n (%)	3 (25)	16 (28.1)	3 (27.3)	0.98
Heart failure n (%)	1 (8.3)	9 (15.8)	2 (18.2)	0.77
Any prior arrhythmia n (%)	6 (50)	23 (40.3)	5 (45.5)	0.67
ICD/PPM n (%)	4 (33.3)	4 (7.0)	2 (18.2)	0.03
**ECG parameters**				
QT (msec)				
At admission	380 (355–430)	360 (340–420)	370 (360–450)	0.23
At discharge^a^	380 (360–415)	360 (320–380)	380 (360–415)	0.08
QTc (msec)				
At admission	425 (385–430)	410 (390–440)	420 (395–420)	0.77
At discharge^a^	380 (370–440)	390 (380–440)	390 (386–425)	0.21
QTc dispersion (msec)				
At admission	95 (85–110)	80 (50–110)	85 (70–90)	0.25
At discharge^a^	70 (50–90)	70 (50–90)	65 (50–75)	0.71

^a^Calculated in 60 patients; values are presented as median (interquartile range) or as n (%).

B, patients with brady-arrhythmia; BT, patients with brady- and tachy-arrhythmia; ICD/PPM, implantable cardioverter-defibrillator/permanent pacemaker; QTc, heart rate-corrected QT interval; T, patients with tachy-arrhythmia.

**TABLE 4:** General features, cardiovascular profile, antiarrhythmic treatment, and ECG measurements in the subgroups of arrhythmic patients with previously diagnosed arrhythmias and with new onset arrhythmias.

	Prior arrhythmias (*n* = 34)	New arrhythmias (*n* = 46)	*p-*value
**General findings**			
Age (years)	76.5 (71–80)	72.5 (63–81)	0.08
Death (%)	26.5	23.9	0.79
Comorbidities	3 (2–5)	3 (2–4)	0.89
Lung computed tomography score	9 (5–13)	8 (5–12)	0.99
Lung ultrasound score	11 (7–14)	14 (8–20)	0.19
**Main cardiovascular morbidities**			
Arterial hypertension (%)	79.4	60.9	0.09
Coronary artery disease (%)	33.3	24.4	0.45
Heart failure (%)	17.6	13.0	0.57
ICD/PPM (%)	29.4	0	–
**Antiarrhythmic drugs**			
Beta blockers (%)	23.5	21.7	0.85
NDCCA (%)	5.9	6.5	0.91
Class I antiarrhythmic drugs (%)	20.6	21.7	0.90
Class III antiarrhythmic drugs (%)	11.8	13.0	0.86
Digitalis glycosides (%)	2.9	4.3	0.74
**ECG parameters**			
QT (msec)			
At admission	0.36 (0.33–0.38)	0.36 (0.34–0.38)	0.53
At discharge^a^	0.36 (0.32–0.40)	0.36 (0.34–0.40)	0.97
QTc (msec)			
At admission	0.42 (0.40–0.46)	0.42 (0.39–0.43)	0.36
At discharge^a^	0.41 (0.37–0.43)	0.40 (0.39–0.44)	0.78
QTc dispersion (msec)			
At admission	95 (80–110)	80 (60–110)	0.23
At discharge^a^	70 (50–80)	50 (50–80)	0.65

^a^Calculated in 60 patients; values are presented as median (interquartile range) or as (%).

ICD/PPM, implantable cardioverter-defibrillator/permanent pacemaker; NDCCA, non-dihydropyridine calcium channel antagonists; New arrhythmias, new-onset arrhythmias; Prior arrhythmias, previously diagnosed arrhythmias; QTc, heart rate-corrected QT interval.

By the univariate analysis, the factors/conditions associated with the risk of arrhythmia in our cohort of patients with COVID-19 were advanced age, chronic kidney disease, chronic obstructive pulmonary disease, systemic arterial hypertension, coronary artery disease, heart failure, and the number of comorbid conditions (Table 5). Furthermore, lower values of glomerular filtration rate and hemoglobin, higher circulating levels of troponin, C-reactive protein, and procalcitonin, and higher values of QTc dispersion were found to be associated with the risk of arrhythmia (Table 5). The multivariate analysis found that factors independently associated with the risk of arrhythmia were age, coronary artery disease, and circulating troponin, whereas an independent association of a prolonged dispersion of QTc length with the risk of arrhythmia was only slight (Table 5).

**TABLE 5:** Univariate and multivariate analyses of risk factors potentially associated with arrhythmia.

	Univariate analysis			Multivariate analysis		
	OR	CI	*p*-value	OR	CI	*p*-value
Age	1.13	1.1–1.2	<0.0001	1.14	1.07–1.22	0.0004
Chronic kidney disease	3.13	1.25–7.89	0.02	0.22	0.02–2.11	0.83
COPD	3.19	1.42–7.15	0.005	1.50	0.43–5.26	0.99
Rare diseases	7.23	0.83–63.15	0.07	–	–	–
Arterial hypertension	2.21	1.21–3.99	0.009	1.09	0.42–2.81	0.29
Coronary artery disease	6.17	2.49–15.32	<0.0001	12.7	2.38–68.01	0.005
Heart failure	21.4	2.73–168.4	0.004	3.34	0.10–112.1	0.54
Any prior arrhythmia	0.86	0.31–2.40	0.77	–	–	–
Number of comorbidities	1.50	1.24–1.82	0.02	0.67	0.35–1.28	0.22
Sodium	1.00	0.97–1.03	0.98	–	–	–
Magnesium	0.20	0.04–0.94	0.04	0.86	0.01–64.04	0.95
Creatinine	1.54	0.87–2.73	0.14	–	–	–
Glomerular filtration rate	0.97	0.96–0.98	<0.0001	1.03	0.99–1.06	0.55
B-type natriuretic peptide	1.003	1.00–1.006	0.02	–	–	–
Troponin	1.07	1.03–1.12	0.0004	1.05	1.003–1.10	0.04
C-reactive protein	1.05	1.01–1.09	0.02	1.025	0.98–1.07	0.98
Ferritin	1.00	1.00–1.00	0.16	–	–	–
D-dimer	1.00	1.00–1.00	0.03	–	–	–
Procalcitonin	2.63	1.22–5.69	0.01	1.310	0.84–2.03	0.30
Hemoglobin	0.82	0.71–0.95	0.009	0.89	0.64–1.21	0.77
LMWH	0.57	0.18–1.76	0.97	–	–	–
QTc dispersion	1.02	1.008–1.04	0.001	1.02	0.99–1.04	0.08

COPD, chronic obstructive pulmonary disease; LMWH, low-molecular-weight heparin; QTc, heart rate-corrected QT interval.

## Discussion

On the basis of the present results, there is evidence of an overall high prevalence (40%) of rhythm disorders in patients with COVID-19, as well as a high incidence (23%) of new-onset arrhythmic events occurring during the in-hospital period. Namely, both the previous and new-onset sustained and non-sustained atrial tachycardia and/or atrial fibrillation, ventricular ectopic beats, significant bradycardia, and atrioventricular or intraventricular conduction disturbances were the main rhythm disturbances mostly recorded on cardiac telemetry in our cohort of patients.

Patients with arrhythmia in the present study were older than those patients without arrhythmia, and showed a trend toward an impaired renal function, a hyperinflammatory/infectious status, an altered coagulative balance, an impaired myocardial propagation of electrical impulse, and subtle myocardial damage, irrespectively of lung degree involvement. Our findings are in line with previously reported analyses in patients with COVID-19, which emphasized a strong association of arrhythmia with augmented circulating levels of BNP and troponin, both consistent with a condition of myocardial injury (16).

Analysis of ECG parameters recorded on admission day, which mostly coincided with the worst phase of the disease, revealed a prolonged duration both of the QT and QTc interval, and higher values of QTc dispersion, in patients with arrhythmia. In addition, it was found, especially in the group of patients with arrhythmia, that values of QTc dispersion tended to decrease as the disease evolved toward healing, which was reached by all the survivor patients at the end of the hospitalization period. Notably, an elongated dispersion of QTc interval represents an expression of a locally inhomogeneous propagation of electrical impulse to myocytes which, on one hand, translates into a heterogeneity of myocardial repolarization, and, on another hand, exposes the individuals to develop arrhythmic events, both fatal and non-fatal, and heart failure secondary to cardiomyopathy (9, 17). To date, the exact mechanisms of prolonged dispersion of QTc interval in our patients with COVID-19 are not completely elucidated. Hypothetically, a SARS-CoV-2-related condition of myocardial tissue inflammation together with subtle impaired myocyte oxygenation both leading to local and small fibrosis could likely offer an explanation for the altered myocardial refractory period documented in our cohort of patients.

At univariate analysis, the risk of arrhythmia was found to be significantly associated with older age, some chronic cardiovascular and non-cardiovascular diseases, including systemic arterial hypertension, heart failure, coronary artery disease, kidney failure, and obstructive pulmonary disease, and increased QTc dispersion in our patients with COVID-19. Similarly, it resulted an association of circulating levels of procalcitonin, C-reactive protein, and D-dimer, as the expression of a combined condition of infectious/hyperinflammatory status together with coagulative imbalance, with the risk of developing rhythm abnormalities among patients of the present study. Altogether, our findings are aligned with the results of previous studies, which found a strong association between a condition of hyperinflammatory status/myocyte injury and adverse in-hospital outcomes, with or without fatal and non-fatal rhythm disturbances (16, 18). In addition, the multivariate logistic regression analysis confirmed an independent potential proarrhythmic role played by age, coronary artery disease, and subtle myocardial damage. In the multivariate analysis, QTc dispersion and the risk of rhythm disorders resulted only slightly associated in our not large series of patients with COVID-19. However, a number of considerations, including significant differences in values of QTc dispersion between patients with and without arrhythmia during the worst phase of the disease, the trend of QTc dispersion values throughout the in-hospital period toward a normalization, especially among patients with rhythm disorders, and an already established proarrhythmic potential for abnormalities in QTc dispersion, led to speculate that an increase in QTc dispersion values has likely played a role in the genesis of rhythm abnormalities in our patients with COVID-19. Moreover, this suggestion has to be taken with caution and further studies designed in larger cohorts of patients are needed to confirm this hypothesis in the future.

Due solely to an episode of ventricular fibrillation, arrhythmia was fatal in one subject among our patients with COVID-19. From a practical point of view, one can hypothesize that arrhythmia likely represents a negative prognostic factor *per se*, irrespectively of the final cause of death. Furthermore, the results of the present study have stated that the type and origin of arrhythmias did not influence in-hospital mortality in our series of patients or whether arrhythmias were already present or not in the patient’s personal history before hospitalization. In fact, by comparing the three subgroups of patients with arrhythmias, which consisted of subjects with tachy-, bradyarrhythmia, or both, and substantially matched for age, clinical general characteristics, lung involvement, cardiovascular *profile*, and ECG parameters, there was evidence of in-hospital mortality not statistically different. In analogy, no significant differences in terms of clinical findings, antiarrhythmic treatment, ECG parameters, and in-hospital outcomes were found by comparing patients with and without arrhythmias diagnosed before hospital admission. Except for domperidone (for a very brief period), no other drugs with a proarrhythmic potential were used in our patients. In line with previous reports (5), the present study has confirmed that rhythm disorders are able to confer a worse in-hospital outcome, with a risk about 40-fold higher for all-cause in-hospital mortality than that documented in patients free of arrhythmia; about 95% of patients deceased during in-hospital period exhibited rhythm disturbance on cardiac telemetric monitoring.

Of note, the in-hospital mortality rate (about 10%) in our COVID-19 center was found to be relatively low as compared to the findings that emerged from other COVID-19 centers during the first COVID-19 pandemic wave. In fact, analysis of patients’ outcomes from some other COVID-19 centers, including Southern and Northern Italy (all-cause mortality ranging between 23 and 38%), United Kingdom (all-cause mortality of 26%), and New York City, United States (all-cause mortality of about 24%), demonstrates unequivocally that in-hospital outcome of our series of patients with COVID-19 is likely better (19–23); the reasons are not easily understandable, but several could be the factors involved. First, the availability of evidence-based recommendations derived from updated scientific data has allowed ameliorating the overall management of COVID-19 (24). Second, our COVID-19 center adopted in each patient an approach similar to that commonly used in the subintensive care unit, which is basically based on continuous telemetric monitoring of vital parameters and ECG trace too. This care management protocol allowed healthcare personnel to promptly identify any arrhythmic event and to intervene appropriately where necessary. For all these reasons, an approach similar to a subintensive care unit should be probably encouraged when organizing the workup in a COVID-19 ward, especially when treating patients with a previous personal history of rhythm disorders and/or with a manifest proarrhythmic profile (25).

On the basis of the present findings, a suggestion could be provided; accurate analysis of ECG, including measurements of length and dispersion (corrected and non-corrected) of the QT interval, could be of some usefulness in the general management of patients suffering from SARS-CoV-2 infection. ECG represents a simple, but essential tool in clinical practice. This is particularly true, especially in some settings of patients, including those affected by COVID-19, in whom a prompt diagnosis of eventual abnormalities in electrical impulse propagation related to subtle myocardial involvement and an accurate prognostic stratification are likely imperative.

### Study limitations

The relatively small sample size represents an important limitation of the present study. Moreover, despite our efforts to precisely collect the patient’s personal history, it is not excluded that previous arrhythmic events, i.e., those that occurred before hospitalization, have been underreported. Unfortunately, echocardiographic examination primarily aimed to evaluate left ventricular ejection fraction, as a surrogate of global cardiac performance, was carried out in a small cohort of patients only, so limiting the possibility to better and further explore the postulated association between myocardial damage and the risk of arrhythmias in our series of patients with COVID-19. However, homogeneity of data and a scanty interobserver variability due to standardization of patients’ observation and treatment protocols probably might reduce methodological bias as above reported. Finally, unlike the majority of previous reports, which were mostly planned as multicenter studies, the present study was conceived as a unicentric study, with the advantage that the healthcare protocol was rigorously standardized, so avoiding a number of confounding factors related to differences in patients’ management protocols from other participants centers.

## Conclusion

The data of the present study confirm a high prevalence of arrhythmia among patients affected by SARS-CoV-2 infection and the predictive role of arrhythmia in defining in-hospital prognosis. Aging, coronary artery disease, hyperinflammatory status, coagulative unbalance, subtle myocardial injury, and elongation of QTc dispersion are all the factors associated with the risk of developing arrhythmias among patients with COVID-19. Hence, routinary use of cardiac telemetric monitoring in a COVID-19 ward seems to offer some advantage in the general management of such patients. Consequently, subjects likely more prone to develop rhythm disorders, especially when aged and/or affected by chronic cardiac comorbidities, should be encouraged to undergo vaccination.

## Member of the Vanvitelli COVID Collaborators

Acquired the data: Abitabile Marianna, Beccia Domenico, Brin Chiara, Carusone Caterina, Cinone Francesca, Colantuoni Sara, Del Core Micol, Gjeloshi Klodian, Imbriani Simona, Macaro Domenico, Medicamento Giulia, Meo Luciana, Nappo Francesco, Padula Andrea, Ranieri Roberta, Ricozzi Carmen, Ruosi Carolina, Sellitto Ausilia, Sommese Pino, Villani Angela, and Christian Catalini. Acquired and analyzed the data: Aprea Concetta, Cirigliano Giovanna, and Ruocco Rachele.

## Data availability statement

The raw data supporting the conclusions of this article will be made available by the authors, without undue reservation.

## Ethics statement

The studies involving human participants were reviewed and approved by Ethic Committee of the University of Campania Luigi Vanvitelli. The patients/participants provided their written informed consent to participate in this study.

## Author contributions

DC designed the research, analyzed the data, and wrote the manuscript. CR acquired the data. RN conducted the observations and analyzed the data. AM provided the reagents and analyzed the data. GU and LR analyzed the data. GC provided the reagents and conducted the observations. LA analyzed the data and contributed to writing of the manuscript. Vanvitelli COVID-19 Collaborators contributed to the data management and writing. All authors contributed to the article and approved the submitted version.
